# The Killer *Saccharomyces cerevisiae* Toxin: From Origin to Biomedical Research

**DOI:** 10.3390/microorganisms12122481

**Published:** 2024-12-02

**Authors:** Carlos Molina-Vera, Verónica Morales-Tlalpan, Amairani Chavez-Vega, Jennifer Uribe-López, Jessica Trujillo-Barrientos, Juan Campos-Guillén, Jorge Luis Chávez-Servín, Teresa García-Gasca, Carlos Saldaña

**Affiliations:** 1 Membrane Biophysics and Nanotechnology Laboratory, Natural Sciences Faculty, Autonomous University of Quéretaro, Av. De las Ciencias S/N, Juriquilla, Querétaro 76220, Mexico; cal.move12@gmail.com (C.M.-V.); vmtlalpan@gmail.com (V.M.-T.); chavezamairani@gmail.com (A.C.-V.); jenniferandreauribelopez@gmail.com (J.U.-L.); jess.trujillo2@gmail.com (J.T.-B.); 2 National Laboratory for Advanced Scientific Visualization (LAVIS-FCN-UAQ), Querétaro 76230, Mexico; 3 Faculty of Chemistry, Autonomous University of Quéretaro, Av. De las Ciencias S/N, Juriquilla, Querétaro 76320, Mexico; juan.campos@uaq.mx (J.C.-G.); jorge.chavez@uaq.mx (J.L.C.-S.); 4 Molecular Biology Laboratory, Facultad de Ciencias Naturales, Autonomous University of Quéretaro, Av. De las Ciencias S/N, Juriquilla, Querétaro 76230, Mexico; tggasca@uaq.edu.mx

**Keywords:** *killer* toxin, coevolution, mechanism of action

## Abstract

The *killer* systems of *S. cerevisiae* are defined by the co-infection of two viral agents, an M virus and a helper virus. Each *killer* toxin is determined by the type of M virus (ScV-M1, ScV-M2, ScV-M28, and ScV-Mlus), which encodes a specific toxin (K1, K2, K28, and Klus). Since their discovery, interest in their potential use as antimicrobial agents has driven research into the mechanisms of action of these toxins on susceptible cells. This review provides an overview of the key aspects of *killer* toxins, including their origin and the evolutionary implications surrounding the viruses involved in the *killer* system, as well as their potential applications in the biomedical field and as a biological control strategy. Special attention is given to the mechanisms of action described to date for the various *S. cerevisiae killer* toxins.

## 1. Introduction

The discovery of *Saccharomyces cerevisiae* strains infected with double-stranded RNA (dsRNA) viruses capable of killing other “sensitive” strains marked the inception of research into *killer* strains [1,2]. It was then demonstrated that only specific yeast strains could secrete protein-based toxins, resulting in lethal effects on sensitive strains. These secreting strains were termed “*killer* yeast”, with the secreted protein referred to as “*killer toxin*”. Subsequent investigations revealed that toxin-secreting strains possess antifungal activity [3,4], and surprisingly, other fungi might also produce similar toxins, such as *Zygosaccharomyces bailii*, *Hanseniaspora uvarum*, and *Ustilago maydis* [5,6,7]. Furthermore, it was discovered that not only do dsRNA viruses confer the coding genes for toxins, but toxins encoded in chromosome and dsDNA plasmids have also been identified [4,8,9,10,11,12].

To date, the primary viruses encoding *killer* toxins in *S. cerevisiae* include the M viruses ScV-M1, ScV-M2, ScV-M28, and ScV-Mlus, each encoding a specific toxin (*K*1, *K*2, *K*28, and *K*lus) and reliant on the coexistence of an L-A helper virus to sustain their replication within the yeast host [13]. These viruses belong to the Totiviridae family, encompassing five viral genera: *Giardiavirus*, *Leishmaniavirus*, *Trichomonasvirus*, *Victorivirus*, and *Totivirus*.

ScV-L-A (*S. cerevisiae* Virus L-A) virions contain a single linear (+) dsRNA molecule ranging from 4.6 to 7 kbp in length. This genome comprises two ORFs: the 5′ ORF encodes the capsid protein Gag, which possesses the ability to bind and remove the 5′-cap of host mRNA via the Gln139-Ser182 region and its interaction with His154 of Gag. This mechanism employs host mRNA as a decoy for the 5′-3′ exoribonuclease, thereby safeguarding the viral genome [14]. The 3′ ORF encodes RdRp (RNA-dependent RNA polymerase) and exhibits ssRNA binding activity, along with capsid structures displaying T = 2* symmetry, predominantly composed of a single coat protein (CP) [15]. The Gag monomer can assume two conformations, A and B, each with significant structural disparities. The capsid consists of 12 AB decamers, wherein each decamer comprises 5 A subunits alternating with 5 B subunits to form a decameric structure [14]. Unlike other viruses, the capsid of ScV-L-A is less densely packed, facilitating enhanced mobility of the template strand and conferring a notable advantage to the virion.

Both L-A and M viruses are regulated by specific factors within the yeast host. The SKI exosome complex, comprising Ski3, Ski8, and the DEVH ATPase Ski2, facilitated by the GTPase Ski7 [16,17,18,19], governs the levels of viruses within the cell [20,21]. Reduced expression levels of these genes have been demonstrated to disrupt viral RNA processes, resulting in the “super *killer*” phenotype [22,23]. Furthermore, these viruses exhibit high sensitivity to alterations in ribosomal structure [22]. Among the MAK (Maintenance of *Killer*) genes, mutations in MAK3 (Gag catalytic N-acetyltransferase subunit), MAK10, and MAK31 genes lead to decreased expression of ribosomal subunit 60, thereby disrupting viral RNA replication processes. These genes constitute the NatC acetyltransferase complex, which acetylates the L-A virus Gag capsid protein and contributes to ribosomal maintenance [24,25,26].

## 2. Evolution of the Family Totiviridae

In recent years, viruses belonging to the Totiviridae family have emerged as a significant model for investigating viral evolution and interactions. It has been revealed that these viruses exhibit a broader host diversity than previously recognized, and the phylogenetic relationships among open reading frames (ORFs) within this family are notably variable. An illustrative example of this variability is UmV-H1, a dsRNA virus hosted by the fungus *Ustilago maydis*. A study demonstrated that its RdRp shows closer phylogenetic affinity to insect dsRNA viruses compared to other viruses within the same family [27]. Furthermore, two new genera have been proposed within the Totiviridae family: Artivirus, which infects arthropods and some fish species, and Insevirus, which primarily infects insects [28]. Moreover, the Totiviridae family displays distinctive genomic elements, such as type 2A sequences. These sequences consist of oligopeptides containing 8 to 9 amino acid “signature” sequences that regulate the ribosome skipping effect. They are analogous to internal ribosome entry site (IRES) sequences, leading to proteinase-independent polyprotein anchor co-translation. Various viruses containing these type 2A sequences express an anchor site situated within the ORF1 region responsible for encoding Gag. Consequently, this sequence has the capacity to encode 2 to 3 distinct proteins besides Gag. Among them is the RNA-binding protein (RBP) harboring a dsRNA binding domain, alongside a small protein 2 (SP2), which is thought to be associated with the cell entry and extracellular transmission mechanisms of infectious myonecrosis virus (IMNV). Although not ubiquitous among all members of the Totiviridae family, these type 2A sequences seem to represent a significant strategy for enhancing and perpetuating genomic complexity, constituting a pivotal aspect in viral evolution [29].

Another remarkable aspect is the family’s formation of a monophyletic group, in contrast to other dsRNA viruses that exhibit polyphyletic characteristics. Its origins have been postulated to occur just prior to the divergence between Fungi and Protozoa. This proposition finds support in the evidence provided by Victorivirus (a mycovirus), which demonstrates closer phylogenetic relatedness to protozoan viruses (Leishmaniavirus and Trichomonasvirus) than to Totivirus (another mycovirus). Phylogenetic analyses focusing on the amino acid variabilities of RdRp and CP further bolster the hypothesis of substantial divergence, attributed to the viruses’ capacity to infect phylogenetically close yet distinct hosts [28]. Furthermore, the notion of a mono-segmented RNA serving as the common ancestor of dsRNA viruses has been proposed, with descendant genomes later becoming segmented. Consequently, the mono-segmented characteristic observed in Totiviridae positions the family at the foundational level of dsRNA virus evolution [30].

The origin of mycoviruses has been a subject of significant interest since their discovery, primarily due to their lack of an extracellular phase [31,32]. Two complementary hypotheses have emerged to explain their origin and evolution: the Ancestral Coevolution theory, suggesting a long-standing process of coevolution between fungi and viruses, and the Phytovirus Hypothesis, proposing that mycoviruses originated from phytoviruses, possibly through transmission from plants to fungi with which they had a symbiotic relationship, either mutualistic or parasitic [33,34,35]. The evolutionary origins of mycoviruses remain ambiguous, leading to uncertainty regarding the diverse range of hosts they can infect across different viral genera. In the case of the Totiviridae family, the hypothesis posits the existence of an ancestral self-replicating mRNA, yet the mechanism by which they initially infected fungi remains unclear. A study involving the *Giardia lamblia* virus demonstrated its ability to enter cells through free virions or transfection via electroporation of positive RNA strands transcribed from the original dsRNA virion [36]. Nevertheless, further research is imperative to unveil the initial infective process underlying virus–fungus interactions.

## 3. The *S. cerevisiae* *Killer* Toxins

### 3.1. The K1 Toxin 

The *K*1 toxin, the original (first) member among *killer* toxins, was the first to be identified. Its expression is closely associated with the presence of the ScV-M1 virus, characterized by a 1.8 kb genome housing a single ORF responsible for the toxicity and immunity conferred by this toxin [37,38]. This ORF encompasses the pptox sequence, comprising four domains—δ, α, γ, and β—each playing crucial roles in toxin processing, infection, and immunity. The processing of the toxin commences with the import of pptox into the endoplasmic reticulum (ER), where γ undergoes glycosylation and disulfide bridges are formed, linking α and β. Subsequent transportation of the glycosylated pptox to the Golgi involves the action of Kex2p endopeptidases, leading to the removal of δ and γ subunits, thus yielding the mature *K*1 toxin in the form of a 36 kDa α-β heterodimer. Ultimately, the mature toxin is secreted outside the cell via vesicles [4,39,40,41]. Mutagenesis studies, involving the elimination of disulfide bridges, validate that the toxin’s toxicity hinges on the structural configuration conferred by sulfur bonds, alongside the presence of Cys within the α-subunit, while the β-subunit proves indispensable for binding to the target cell [38,42,43,44]. Although the crystallographic structure of either *killer* toxin is not currently available, in silico and mutagenesis models of *K*1 based on Cys indicate that the toxin remains stable due to a disulfide bridge between C92 and C239. The remaining Cys residues of both subunits may exist as free thiol groups or participate in stabilizing intramolecular disulfide arrangements. Notably, C95 and C107 in the α-subunit are implicated in immunity, while C248 and C312 are responsible for binding to intact cells and spheroplasts [42]. These findings complement the proposed immunity and toxicity mechanisms, wherein the toxin enables yeast to self-protect from its lethal effects [45,46]. While the proposed models and data provide insight into the intramolecular structure and interactions of *K*1, the lack of a three-dimensional model precludes a definitive determination of which residues are free or responsible for maintaining *K*1 in its final structure.

### 3.2. K2 and Klus Toxins

In contrast to the well-studied *K*1 toxin, the *K*2 toxin remains poorly understood. Similar to its counterparts (*K*1, *K*28, and *K*lus), this toxin is associated with the presence of a toxin-encoding virus. Specifically, in the case of *K*2, it is linked to the ScV-M2 virus and the helper virus L-A-2. The processing of the preprotoxin (pptox) is conserved, resulting in the secretion of the mature form composed of a 36 kDa α-β heterodimer [47,48]. Interestingly, its structure differs from *K*1 and *K*28 by the absence of a spacer subunit between the α and β subunits, retaining only the signal peptide [49]. Recent studies have demonstrated that the N-terminal sequence of the pptox, in addition to its signaling function, contains a 47 amino acid signal that is both necessary and sufficient to confer immunity to the *K*2 toxin [50]. However, the mechanism by which this peptide counteracts *K*2 activity remains unclear.

The *K*lus toxin remains a poorly studied agent in terms of its structure and mechanism of action following its recent discovery in 2011 from wine-isolated strains [51]. Similar to its counterparts, the ORF at the N-terminal end contains a signal peptide sequence, as well as cleavage sites for the Kex1p and Kex2p peptidases (Lys-Arg- and Arg-Arg- cleavage in the immature protein) and N-glycosylation sites [52]. Interestingly, the ORF of the preprotoxin is related to the chromosomal gene YFR020W (32% identity and 51% similarity), whose function is unknown. However, this relationship opens an intriguing research avenue regarding the evolution of this virus and its co-evolution with the yeast host [53,54,55]. Disulfide bond prediction studies indicate two potential binding sites within the C-terminal domain, predicting a putative 144 aa β-structure. However, the structure of this toxin remains unknown.

### 3.3. K28 Toxin

The first reports on the *K*28 toxin were published in the late 1980s and early 1990s, identifying it as a toxic agent of the same family as the *K*1 and *K*2 toxins [56], whose expression is determined by the presence of the M28 virus and L28 helper virus [57]. Structurally, both *K*1 and *K*28 maintain similarities in their arrangement of domains that make up the preprotoxins (pptox) of both, conserving the prepro region in the N-terminus, which is necessary for the toxin’s secretion system [58], followed by the α (10.5 kDa), γ (containing N-glycosylation sites and recognition sites for Kex1p and Kex2p), and β (11 kDa) subunits, and finally an HDEL retrograde transport sequence. It has been shown experimentally that the mature form of *K*28 is maintained as a 21 kDa heterodimer at pH less than 6, stabilized by a disulfide bridge between C56 of the α subunit and C333 of β. Interestingly, upon increasing pH, the mature toxin can form agglomerates of trimers and tetramers due to the interactions of the three additional cysteines found in β (C292, C307, and C340), which are involved in the toxicity of the α/β dimer [59,60].

## 4. The Mechanisms of Action of *Killer* Toxins

### 4.1. The Mechanism of Action of K1

Each of the described *Killer* toxins possesses unique mechanisms of action. To date, the *K*1 toxin has been the most extensively studied among all *Killer* toxins. Its toxicity results from the interaction of the mature toxin with the target site of a sensitive cell. However, the exact mechanism of action remains unclear, with multiple scenarios proposed.

The first mechanism described for elucidating *K*1 activity occurs in two phases. Initially, the mature toxin binds to a 1,6-β-D-glucan receptor on the yeast cell wall [1]. This receptor is ubiquitous in both immune and sensitive yeast strains, and its expression requires the involvement of *K*RE (*Killer* Resistance) genes involved in cell wall biosynthesis [44,61]. In the second phase, the mature α/β toxin increases potassium ion flow across the plasma membrane, leading to cell death [62,63]. Most *K*RE mutants do not interact with the toxin and are thus resistant to *K*1 action, whereas spheroplasts are sensitive to the toxin. This suggests that *K*1 action may be independent of the cell wall and primarily associated with the plasma membrane.

The second proposed mechanism indicates that the *K*1 toxin causes uncontrolled efflux of potassium, protons, ATP, and other small molecules from target cells through pore formation [38,63]. This hypothesis is supported by experimental mitigation of the *killer* effect, such as the reduction in *K*1 activity by administering high levels of external potassium, which protects sensitive cells from *K*1 action [64]. Additionally, spheroplasts and reconstituted liposomes exposed to *K*1 are depleted entirely of potassium [65].

The third mechanism was discovered incidentally during the sequencing of the *S*. *cerevisiae* genome. Genes encoding proteins with structural properties, including the Tok1p channel, were identified. Tok1p is an outward-rectifying potassium channel with two tandem pore domains located in the yeast plasma membrane [66,67,68]. The presence of two tandem pore domains led to various hypotheses about their function, ultimately demonstrating that both pores are crucial for selectivity and work together to form a single potassium-permeation pore [69]. Interestingly, the *K*1 toxin can activate Tok1p channels in sensitive strains, spheroplasts, and *X. laevis* oocytes expressing the channel [70,71,72]. Experiments showed that the absence of the channel confers resistance to the toxin, while overexpression increases sensitivity. The action of the *K*1 toxin on the Tok1p channel of *S. cerevisiae* occurs in two main phases: (1) In the initial interaction with the Tok1p channel, the mature *K*1 toxin binds to the Tok1p potassium channel on the plasma membrane of a sensitive cell, activating the channel. This binding leads to increased potassium ion outflow from the cell, creating plasma membrane depolarization. (2) During depolarization and cell death, the potassium loss results in depolarization of the plasma membrane and a disruption in cellular homeostasis. This mechanism is essential for *K*1 toxicity, as uncontrolled ion flow disrupts normal cellular functions, ultimately causing cell death [70].

**Figure 1:**
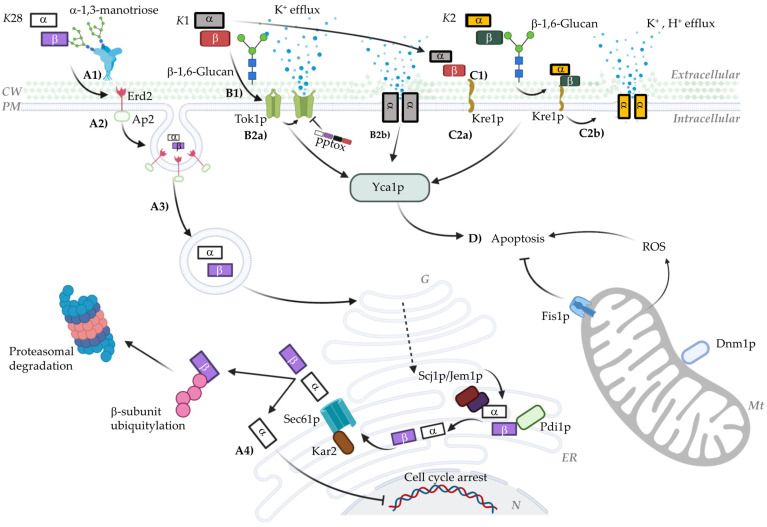
Model of the mechanism of action of *Killer* toxins. This graphical representation shows the different proposed mechanisms of action for the *K*1, *K*2, and *K*28 toxins. (**A1**–**A4**) The transit of the *K*28 toxin, beginning with the recognition of α1-3 mannose trimers of the cell wall mannoproteins, followed by binding to the Erd2 receptor in an energy-independent process. Once inside the cell, the toxin is transported via retrograde transport to the ER, where it is linearized and transported to the cytosol via the Sec61p complex. In the cytosol, the α subunit enters the nucleus, arresting the cell cycle at G1/S, while the β subunit is polyubiquitinated and degraded in the proteasome. (**B1**) The recognition of the *K*1 toxin by the β-1,6-Glucan receptor, followed by recognition of the Tok1p channel (**B2a**), destabilizing it and causing uncontrolled K^+^ release. It also shows recognition by the *K*re1p receptor (**B2b**), described as the primary receptor for this toxin. After recognition, the α subunit aggregates in the cell membrane, forming a cationic channel. Similar to *K*1, the *K*2 toxin shares the β-1,6-Glucan and *K*re1p receptors (**C1**,**C2a**). The toxin is subsequently translocated to the membrane (**C2b**), where its ionophoric action destabilizes the membrane, causing its rupture. (**D**) The non-canonical pathway of PCD in response to low toxin concentrations. The internalization of the *K*28 toxin and the action of *K*1 and *K*2, in addition to deregulating the cell cycle and membrane, respectively, trigger the activation of the Yca1p caspase, as well as Dnm1p activity and ROS accumulation. Created with Biorender.com.

Moreover, Sesti et al. [72] proposed the self-immunity mechanism of the *K*1 toxin in the *S. cerevisiae* Tok1p potassium channel. According to their study, *K*1 preprotoxin (pptox) interacts with Tok1p, causing an internal blockade that interferes with the channel’s function, ultimately conferring immunity to the cell against the toxin. The proposed mechanism suggests that *K*1 pptox acts from within the cell, interacting with the internal side of the Tok1p channel and blocking potassium ion flow through it. This interaction prevents the mature toxin, which acts externally, from exerting its lethal effect on the plasma membrane. To clarify, pptox provides a protective effect by occupying the Tok1p channel from the inside, avoiding pore formation or the potassium flux alteration that the toxin might induce in sensitive cells. This internal blocking mechanism by pptox explains the natural immunity some cells exhibit against the *K*1 toxin and underscores the significance of Tok1p in the action of this *killer* toxin. The research further demonstrates that strains lacking Tok1p are resistant to *K*1, while those overexpressing this channel show increased sensitivity. This model suggests that the action of *K*1 in sensitive cells heavily relies on the availability and functionality of Tok1p in the plasma membrane [72]. Considering the recognition of the mature toxin by the 1,6-β-D-glucan receptor via the β subunit (Figure 1(B1)), it is hypothesized that the molecular mechanism occurs in two phases: the β subunit initially recognizes this receptor through cysteine 248 [42,43], followed by the α subunit interacting with the Tok1p channel (Figure 1(B2a)). However, the specific molecular interaction between *K*1 and Tok1p remains uncertain.

The fourth and final proposed mechanism presents a viewpoint contrary to the binding of the *K*1 toxin to the Tok1p potassium channel. It is based on the observation that the tok1-null strain does not show significant changes in sensitivity or immunity to the toxin [61]. Alternatively, the *K*re1p receptor located on the yeast cell surface is proposed as the primary binding agent for the toxin [73]. Evidence of its interaction with K1 led to an alternative model where the β subunit of the toxin interacts with its primary receptor, 1,6-β-D-glucan, on the cell wall, followed by translocation and binding to the Kre1p receptor [61]. The importance of this receptor is linked to 1,6-β-D-glucan synthesis, as Δkre1 strains reduce its production by 40% [44,74]. Moreover, these mutants exhibit complete resistance to *K*1, leading to a new molecular mechanism based on Kre1p’s interaction with *K*1, subsequently forming cation-selective pores that cause uncontrolled potassium efflux and proton influx (Figure 1(B2b)) [38,40,45,61,63]. However, recent studies suggest that resistance to *K*1 may not be entirely related to the *K*1–*K*re1p interaction but rather to Kre1p’s role in 1,6-β-D-glucan synthesis [74]. Δ*k*re1 mutants have reduced 1,6-β-D-glucan receptor levels, conferring a *K*1-resistant phenotype [75]. Thus, the exact molecular mechanism still requires experimental validation.

### 4.2. The Mechanism of Action of K2 Toxin

Although the *K*2 toxin has not been extensively studied, key events in its mechanism of action have been determined. It is accepted that *K*2 operates through a two-phase killing system and that its preprotoxin processing is similar to that of *K*1 and *K*28 [76]. However, *K*2 has distinctive action profiles. One notable similarity is the primary recognition of the target cell. Like *K*1, *K*2 uses the 1,6-β-D-glucan receptor and *K*re1p [77,78,79] to subsequently induce physiological changes that trigger cell death (Figure 1(C2a,b). It is in this stage that the two toxins differ. This specific difference allows them to kill each other; that is, although strains are immune to their own toxin, *K*2-producing strains can kill *K*1-producing strains and vice versa [78,80].

Various studies have focused on determining the particularities that differentiate the mechanisms of these two toxins. It has been discovered that *K*2 operates optimally at a pH of 4 [81,82], causing effects on the cell surface such as ruptures and undulations of the cell wall due to disruption of the electrochemical gradient [83]. This event may be due to the ionophore activity of the toxin. The uncontrolled influx of sodium, combined with the efflux of potassium and protons, activates a series of stress signals, cell integrity pathways, increased oxygen consumption, and activation of the HOG pathway and phosphoinositide signaling, which have not been detected in response to K1 [48,50,84].

### 4.3. The Mechanism of Action of K28 Toxin

Unlike the previously described toxins, *K*28 has a unique mode of action as it targets the cell cycle rather than the cell surface or membrane. Its molecular mechanism is more complex compared to other toxins. However, preprotoxin processing is conserved among *killer* toxins, resulting in a 21 kDa α/β heterodimer (10.5 kDa and 11 kDa) [59,85]. The recognition and internalization of the toxin are mediated by the β subunit, involving passage through the cell wall by binding to a 185 kDa mannoprotein that recognizes α1-3 and/or α1-2 mannotriose chains, followed by binding to the membrane receptor Erd2p via recognition of the C-terminal HDEL sequence of the β subunit (Figure 1(A1)) [86,87,88,89]. Subsequently, the toxin is transported from the plasma membrane into the cell through a unique process involving mono-ubiquitination by the Uba1p (E1), Ubc4p (E2), and Rsp5p (E3) proteins, supported by the AP2 complex, allowing endocytosis and retrograde transport through the Golgi apparatus and endoplasmic reticulum (ER) (Figure 1(A2,A3)) [45,90,91,92]. This step is crucial as the α/β toxin complex is linearized by Pdi1p to ensure its transport out of the ER [60]. Subsequently, in the cytosol, the β subunit is ubiquitinated and directed to the proteasome, while the α subunit passively diffuses into the nucleus. Once inside the nucleus, the α subunit interacts with nuclear proteins related to cell cycle control, specifically arresting the cell in the G1/S phase (Figure 1(A4)) [91,93,94,95].

### 4.4. The Mechanism of Action of Klus Toxin and Non-Canonical Pathways

The *K*lus toxin is the most recently discovered, and its mechanism of action remains unknown. However, this toxin can *k*ill *K*1, *K*2, and *K*28 strains while being immune to its own toxin (a common trait among all). A remarkable characteristic of Klus is its less aggressive effect compared to other toxins, with an optimal temperature range of 28–30 °C and pH 4–4.7 [51]. It is also one of the least prevalent under natural conditions [96]. Despite this, the mechanism by which it causes cell death in sensitive strains is still unknown.

Recent studies have uncovered alternative events to the main proposed mechanisms. These scenarios can be considered as either alternative or simultaneous. Programmed cell death (PCD) in yeast has been shown to be an event triggered by interaction with *killer* toxins. Apoptosis, a highly regulated mechanism, can be triggered by numerous effectors, most notably reactive oxygen species (ROS) and caspases [45]. Viral *killer* toxins have been observed to have two pathways of action: (i) a canonical pathway (as described above) and (ii) a secondary pathway (Apoptosis) [97]. High toxin concentrations lead to the canonical pathway, while low concentrations can trigger apoptotic signaling (Figure 1D). For the *K*1 and *K*2 toxins, PCD has been hypothesized as a response to their interaction with plasma membrane receptors (Tok1p and *K*re1p) and intracellular components such as yeast caspase 1 (Yca1p) and mitochondria [98,99,100]. Increased ROS and the pro-death factor Dnm1p (involved in mitochondrial fission) are the main effectors of PCD, while the mitochondrial pore-forming protein Fis1p inhibits the apoptotic signal [101,102,103,104]. This pathway is significantly slower than the canonical pathway (5.5 times slower). However, for the *K*28 toxin, the apoptotic pathway is three times faster than its respective canonical pathway [97]. This variability is essentially due to differences in the mechanisms of action of the two toxins; while *K*1 acts as an ionophore, *K*28 arrests the cell cycle in G1/S, requiring sensitive cells to be in this phase.

## 5. The Role of *Killer* Toxins in Heterologous Expression Systems

Heterologous expression systems have facilitated the manipulation and expression of genes in organisms from different species or cell lines than the original source. These systems enable high production rates of the protein, as well as modifications in the peptide sequence that are not present in its natural source [105]. Eukaryotic organisms, such as filamentous fungi [106], insect cells via baculovirus infection [107,108], plants [109,110], and mammals [111], represent attractive models for studying recombinant proteins in cases where the use of bacteria like *E. coli* is not entirely viable [112]. The heterologous expression system provides an attractive ease of genetic manipulation and the ability to produce large quantities. In the case of yeasts such as *S. cerevisiae* and *Pichia pastoris*, among others, their use as an expression system is suitable for expressing proteins with therapeutic applications and for functional and structural studies [113,114].

In addition to the mentioned species, other *killer* toxin-producing yeasts like *Kluyveromyces wickerhamii*, *Wickerhamomyces anomalus*, and *Tetrapisispora phaffii* have proven effective for use as antimicrobial agents against fungal infections [115,116], biological control of plant pathogens [117], regulators in food safety and quality [118,119,120], and biofuel production [121]. Their recombinant production is ensured through the heterologous expression of the toxins produced by these yeasts. However, it is necessary to clarify the advantages and disadvantages offered by each expression system according to the application needs. While *S. cerevisiae* and *P. pastoris* currently have the most available resources for use as heterologous expression organisms, other yeasts like *Kluyveromyces lactis* and *Yarrowia lipolytica* are emerging as promising candidates in this field [122].

One of the most important aspects of recombinant protein production is ensuring protein secretion. This process depends entirely on its post-translational and/or co-translocation into the lumen of the endoplasmic reticulum (ER), followed by importation into the Golgi. Thus, recombinant protein designs need signal peptides that ensure Golgi importation and subsequent secretion. In the case of S. cerevisiae, one such heterologous expression design involves the use of the *K*28 toxin prepro sequence, which has proven to be a significant element in ensuring the importation and processing of heterologous proteins into the Golgi, ensuring the secretion of the recombinant peptide into the extracellular medium [58,123]. This strategy allows the use of different varieties of *killer* toxins as post-translational regulation elements, ensuring the processing of recombinant proteins.

Recently, various *killer* toxins from *S. cerevisiae* have been recombinantly expressed for various purposes. One example involves the fusion of the *K*28 toxin with fluorescent proteins in *P. pastoris* [124]. This chimeric protein showed proper production and secretion while maintaining its unaltered toxicity. Thus, such toxins fused with fluorescent markers can be utilized for analyzing toxin interactions with different molecular targets. While this strategy can be replicated to study various yeast *killer* toxins, it is essential to determine that their mechanisms of action, structure, and physicochemical properties differ. Hence, the use of such systems should be evaluated case by case.

## 6. The Effect of *Killer* Toxins in the Biomedical Field

Initial evidence of the effect of *S. cerevisiae* toxins was observed in strains of the same species, which were categorized as “sensitive” because they lacked resistance mechanisms against the producing yeast’s toxins [41,72]. Later studies demonstrated that toxin-producing strains have a mechanism that makes them immune to their own toxin, although this immunity does not extend to other toxins [46], and their effect seemed directed at cell wall components. Consequently, the effect of these toxins was evaluated against various yeast-like fungi such as *Pneumocystis carinii*, *Candida albican*s, and *Candida glabrata* [3,116,125]. A recent study showed that *C. albicans* contains a homologous gene to the Tok1p potassium channel (one of the possible *K*1 toxin-binding sites), demonstrating that the presence of Tok1p is related but does not determine sensitivity to the *K*1 toxin in this yeast [71]. Furthermore, it has been determined that the lethal effects of *killer* toxins can be enhanced when two toxins with similar mechanisms of action, such as *K*1 and *K*2, are used synergistically, showing a greater effect when used together [126]. Additionally, it has been demonstrated that the K28 toxin has a toxic effect on strains of *Trichophyton rubrum* and *Microsporum canis* [127], supporting the potential use of *killer* toxins as potent antifungal agents. The observed effects in these yeasts also enabled the use of *killer* toxins as an alternative biotyping method for identifying various strains of *Candida, Nocardia, Debaryomyces,* and *Kluyveromyces* [128,129,130,131,132].

Interestingly, the effect of *killer* toxins is not restricted to fungi; they have also been shown to inhibit pathogenic bacterial strains. Various studies have demonstrated that protein fractions and secondary metabolites from different yeasts such as *S. cerevisiae*, *Pichia, Kluyveromyces lactis, Saccharomyces unisporus, Metschnikowia pulcherrima*, and *Kluyveromyces marxianus* can inhibit the growth of Gram-negative and Gram-positive pathogenic bacteria (*Escherichia coli, Klepsiella. pneumoniae, Listeria monocytogenes, Proteus mirabilis, Pseudomonas aeruginosa, Staphylococcus aureus*, and *Salmonella typhimurium*) [115,133,134,135,136,137]. However, these studies only demonstrate the effectiveness of protein and metabolic fractions without specifying which toxins are being secreted into the medium or which target sites are susceptible to the *killer* effect.

The *killer* toxin produced by *Saccharomyces cerevisiae*, particularly the *K*1 toxin, has shown great potential as an alternative to antibiotics due to its ability to inhibit a variety of microbial pathogens. This protein toxin acts, among other mechanisms, by creating pores in the cell walls and plasma membranes of susceptible organisms, leading to cell death. Thanks to its specific mode of action, *K*1 is particularly effective against certain fungal and bacterial pathogens, positioning it as a potential antifungal and antibacterial tool in biomedical applications.

In addition to inhibiting other strains of S. cerevisiae, the *K*1 toxin has demonstrated efficacy against pathogenic bacteria. Its mechanism of action involves targeting and binding to specific cell wall components, causing membrane depolarization, and disrupting cellular homeostasis. The use of the *K*1 toxin and similar *Killer* toxins could help reduce dependence on conventional antibiotics, particularly for resistant strains. However, additional studies are essential to fully understand the specificity of its action and the immune response it may provoke in different organisms, which is crucial for ensuring its safety and efficacy in therapeutic applications.

Future research will focus on optimizing *Killer* toxin formulations, identifying specific binding sites on target cells, and evaluating the effects of combining *K*1 with other antimicrobial agents. Although this research is still in its early stages, it represents a promising advance toward the development of new antimicrobial agents that could complement or even replace traditional antibiotics, especially in treating multidrug-resistant infections.

## 7. Perspectives

*S. cerevisiae killer* toxins have formed an intriguing field of research encompassing various areas of analysis, from studying the origin and evolution of the viruses responsible for the *killer* phenomenon to modeling the molecular interactions of toxins with their molecular targets. Although there is extensive information on *killer* toxins, many enigmas remain in this model of study. Among the main areas of opportunity in studying *killer* toxins is the determination of their three-dimensional structural elucidation. While prediction models like AlphaFold and AlphaFold2 have made significant advances with the help of artificial intelligence [138], in silico approaches do not reflect the post-translational modifications experimentally determined [40,46]. Additionally, the possibility of establishing molecular dockings with hypothesized binding sites for each toxin is low. Future studies should focus on resolving the molecular structures and elucidating the specific and shared target sites among different microorganisms. Since the prospect of using *killer* toxins is of biomedical and biotechnological importance, it is necessary to determine these mechanisms more specifically to ensure the effectiveness and safety of using *killer* toxins for various purposes.

## Data Availability

The data presented in this study are available upon request.
